# Technical Refinements for Reducing Reoperations in Single-Stage Augmentation Mastopexy: A Retrospective Matched Cohort Study

**DOI:** 10.1007/s00266-024-03917-2

**Published:** 2024-03-11

**Authors:** Matteo Marino, Mario Alessandri-Bonetti, Riccardo Carbonaro, Francesco Amendola

**Affiliations:** 1 Private Practice, Milan, Italy; 2 University of Milan, Via festa del perdono, 7, 20122 Milan, Italy

**Keywords:** Mastopexy, Augmentation mastopexy, Breast implant, Augmentation mammoplasty, Inverted T mastopexy

## Abstract

**Background:**

The goals of mastopexy differ significantly from those of augmentation mammoplasty. Mastopexy is designed to lift and reshape the breasts, while augmentation mammoplasty is designed to increase the volume of the breasts. This conflict causes that one-stage augmentation mastopexies showed a revision rate from 8.7 to 23.2%. The aim of our study is to present some technical refinements for reducing the risk of implant exposure and reoperation.

**Methods:**

We designed a retrospective matched cohort study, including 216 consecutive patients, undergone augmentation mastopexy between January 2013 and December 2022. We divided them in two groups: Group A undergone an inverted-T superomedial pedicled augmentation mastopexy and Group B undergone our inverted-T modified augmentation mastopexy. The groups were matched for clinical and surgical variables, with the surgical technique the only difference between the two.

**Results:**

Complications were registered in ten patients (9.3%) in Group A (two wound breakdowns at T with implant exposure and eight wound dehiscences), six of which required surgical revision. In contrast, only three patients (2.8%) in Group B reported a complication, which was wound dehiscence without implant exposure in all cases. None of the dehiscence required surgical revision. The difference between complication and revision rates was statistically significant.

**Conclusions:**

Separating the implant and the mastopexy dissection planes reduces the implant exposure and the reoperation rate in one-stage augmentation mastopexy.

**Level of Evidence III:**

This journal requires that authors assign a level of evidence to each article. For a full description of these Evidence-Based Medicine ratings, please refer to the Table of Contents or the online Instructions to Authors https://link.springer.com/journal/00266.

**Supplementary Information:**

The online version contains supplementary material available at 10.1007/s00266-024-03917-2.

## Introduction

As outlined by Spear [1] and later remarked by Lee [2] and Sanniec [3], the goals of mastopexy differ significantly from those of augmentation mammoplasty: the former is designed to lift and reshape the breast, reducing the surface of the skin envelope, while the latter increases the breast volume, counteracting the surface reduction of the mastopexy. Additionally, the amount of scarring associated with augmentation mammoplasty is typically minimal, whereas mastopexy generally requires larger and less concealable scars. These differences account for a revision rate as high as 23% [4–10] in one-stage augmentation mastopexy.

Detailed surgical planning, a stepwise approach, and meticulous intraoperative techniques are the key points to minimize the risk of complications and to enhance the results. Nevertheless, implant exposure with subsequent removal represents the most serious local complication, and it is often exacerbated by the wound dehiscence usually presenting at the T-junction in classic inverted-T mastopexy.

This study describes the outcomes of a modified technique for single-stage augmentation mastopexy used for over ten years. We retrospectively compared two single-operator cohorts of patients: one undergoing a classic inverted-T with superomedial pedicle augmentation mastopexy and the other undergoing a modified inverted-T with an extended glandular pedicle augmentation mastopexy in which the glandular flaps are designed to protect the implant from exposure and lower the complication rate. The technique consists in the combination of the subcutaneous glandular dissection performed in vertical [11] and peri-areolar [12, 13] mastopexy, the inferior dermo-glandular flap [14, 15] and the classic approach for augmentation mammoplasty with an incision at the inframammary fold.

## Materials and Methods

We retrospectively analyzed consecutive patients undergone augmentation mastopexy between January 2013 and December 2022. All patients were evaluated, planned, and operated on by the same surgeon (M. M.). Every patient signed a detailed and personalized informed consent prior to the operation.

When deciding if mastopexy was needed in addition to implant placement, we typically assessed the position of the nipple-areola complex (NAC) with the arm raised to 90°. Abduction mimics the expected lift from a simple implant placement. If the NAC was less than 1 cm lower than the ideal new position, we planned a circular areolapexy. Therefore, patients undergone circular areolapexy were excluded from the study. If the NAC was more than 1 cm lower than the expected position, we planned an inverted-T mastopexy.

The patients were divided into two groups: Group A, patients receiving augmentation mastopexy following the classic inverted-T technique with a superomedial pedicle; and Group B, patients treated with the personal technique described below. Each implant was placed in a partial, submuscular, dual-plane pocket. The choice between a round or shaped implant was based on the patient’s preferences regarding upper pole fullness and global appearance of the breast. The degree of breast ptosis was evaluated using the Regnault scale [16]. The follow-up protocol was equal for all included patient's drains removal at 2–3 days after surgery and follow-up visits at 7, 15, 30, 180, and 360 days follow-up. The personal technique of the Group B was introduced in patients operated from February 2017, in the attempt of reducing the implant exposure in case of dehiscences at the T-junction.

Demographic and operative data were retrospectively collected from a prospectively maintained database. Patient demographics included age, body mass index, smoking status, history of previous breast surgery. The primary outcomes were overall complications and implant failure rates. The secondary outcomes were infections, wound dehiscences, and capsular contractures rates. The two groups were matched on degree of ptosis, comorbidities, and implant volume. This study was conducted in accordance with the Declaration of Helsinki.

### Surgical Technique

With the patient standing, the midline, inframammary fold, and breast meridian are marked. The distance between the sternal notch and nipple is marked. The ideal position of the superior border of the nipple-areola complex (NAC) is then marked on the breast meridian, and the distance is checked for symmetry. A point is marked 5 cm inferior to this point and 2 cm from each side to obtain the three reference points for designing the dome of the keyhole. The markings were then continued at the inferior pole for conservative skin excision. With the arms abducted and skin under tension, the inferior pole height was marked at 7.5 cm and an inferior horizontal excision was planned. The implant was then chosen based on the breast base; projection and volume were adjusted according to the patient′s desire. The NAC position on the most projected point of the implant was considered a fixed point, and the level of the IMF was modified accordingly. (Video 1, Supplementary Digital Content 1).

**Fig. 1:**
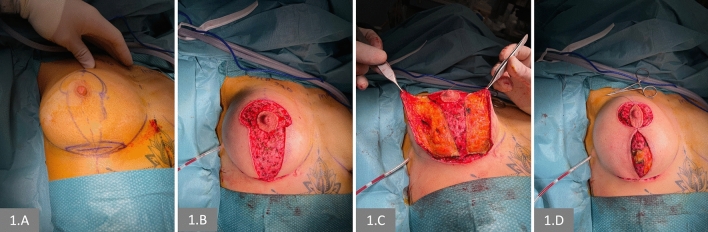
Intraoperative details. **A** The inframammary incision for the implant placement is set at the upper half of the excisional horizontal area in the mastopexy design. **B** Complete de-epithelization is performed after the implant has been placed and its access closed. **C** The medial and lateral pillars and elevated in a subcutaneous, supra-glandular plane, similarly to that followed during a mastectomy. **D** The NAC is lifted, and the pillars are sutured

Intraoperatively, under general anesthesia and with the patient in the supine position, an inframammary incision was placed in the upper half of the planned horizontal lozenge of the inverted-T (Figure 1A). The subpectoral plane was addressed using Ellis′s retractors and blunt elevation with closed Metzenbaum scissors. A dual-plane type II breast pocket was prepared, with soft tissue dissection performed using monopolar electrocautery. A silicon drainage was placed in each pocket, gloves were changed, and the implant was inserted. Every implant received a triple antibiotic immersion prior to its insertion [17]. The lower-pole glandular flap was then sutured to the deep fascia at the level of the new IMF to close the pocket and avoid future dislocation.

Full de-epithelization was performed in the area marked preoperatively (Figure 1B). The medial and lateral pillars were then subcutaneously dissected, following the same plane as the nipple-sparing mastectomy (Figure 1C). After full dissection and closure of the upper and lower areolar borders, the dermal strip of the lower pole was incised to consent to the lifting of the NAC (Figure 1D, Video 2 supplementary Digital Content 2). Eventually, the inferior T-point between the inferior pole and IMF was sutured, and all incisions were closed with monofilament absorbable subcutaneous stitches. A mild elastic dressing is applied.

### Statistical Analysis

Descriptive statistics were used for demographics, and surgical outcomes data among groups were recorded as frequencies and percentages for categorical variables and as means and standard deviations for numerical variables.

For numerical variables, the *T*-test was utilized. For categorical variables, differences were measured using the Chi-square and Fisher exact tests. The analyses were performed using SPSS statistics software (IBM, 1 New Orchard Road, Armonk, New York 10504-1722, United States).

## Results

A total of 216 female patients were included in this study, 107 in group A and 109 in group B. Median age (28 versus 25 years, respectively) and number of active smokers (9.5 versus 10.5 sig/die, respectively) were not significantly different between the groups (*p* > .05). No comorbidities were present in the two groups. Most patients in both groups had Grade III breast ptosis (*n*. 86 versus 77, respectively), without a significant difference. Anatomic implants were used in 86 (80%) patients of the Group A and 87 (79%) patients of the Group B, with a mean volume of 290 cc (± 39) in Group A and 300 cc (± 44) in group B. The median nipple-to-fold length passed from 12 cm preoperatively to 8 cm postoperatively.

The average breast lift was 6 cm for Group A (from a median sternal notch-to-nipple distance of 25 cm to 19 cm) and 5 cm for Group B (median 25–20 cm). The overall average follow-up period was 45 ± 15 months, with no significant difference between the groups (46 months for Group A versus 43 months for Group B, *p* = .17).

**Fig. 2:**
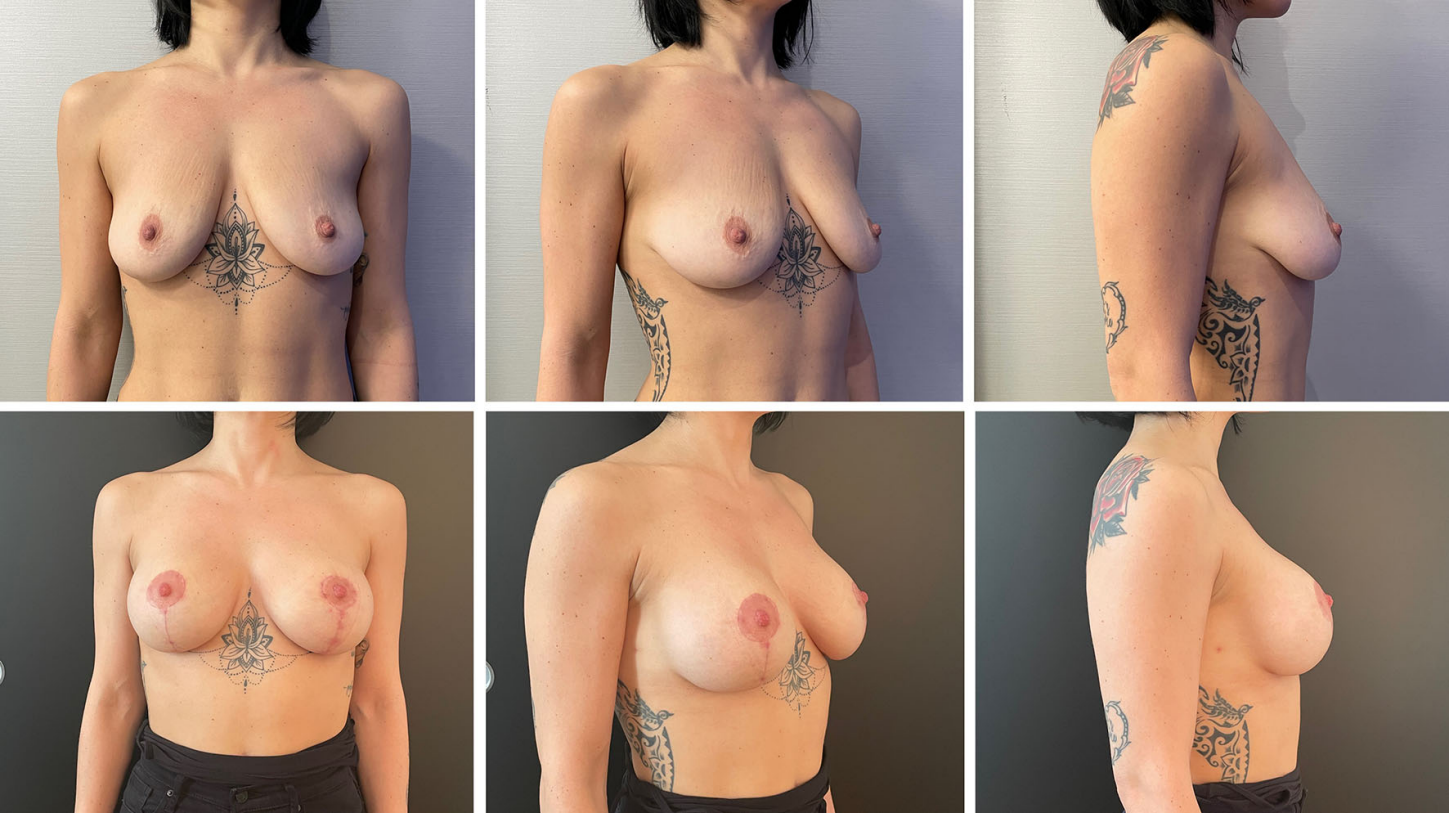
Preoperative (above) and postoperative results at day 30 (below) of the case presented in figure 1

**Fig. 3:**
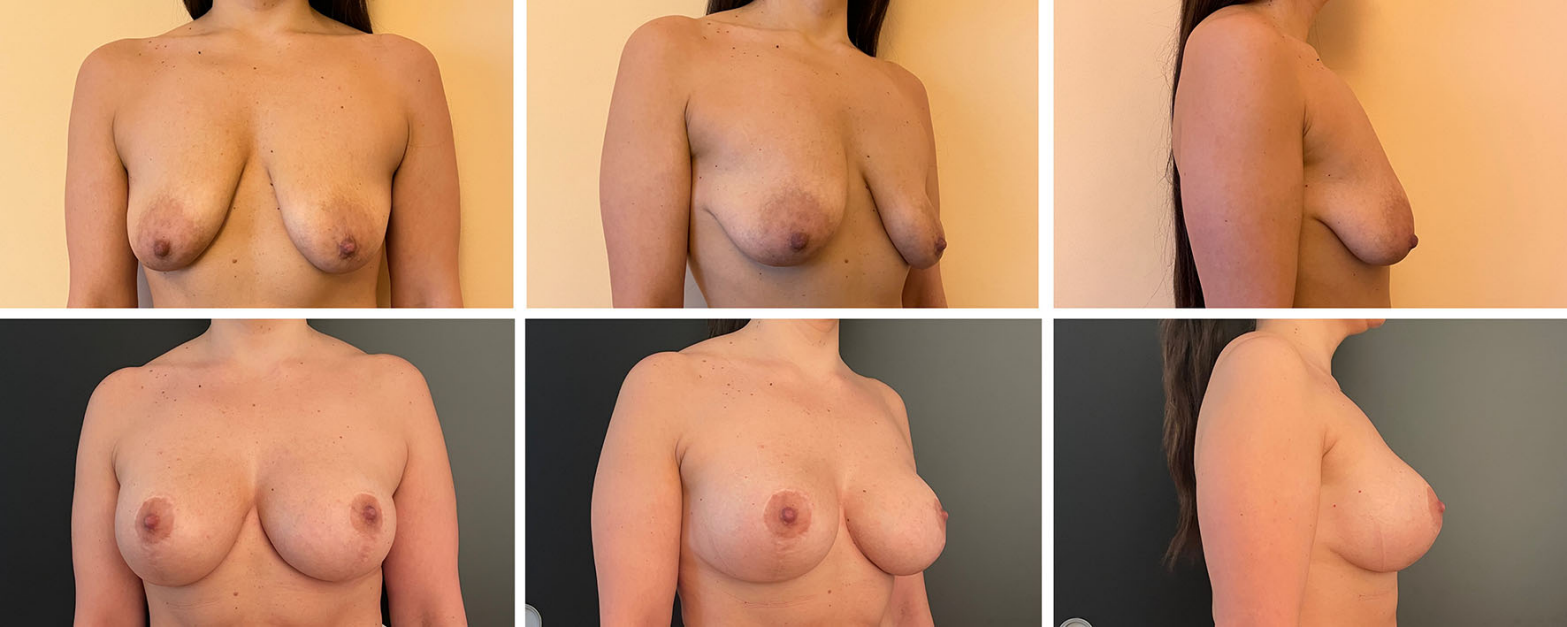
Preoperative (above) and postoperative results at one year (below) of a sample case for Group A

**Fig. 4:**
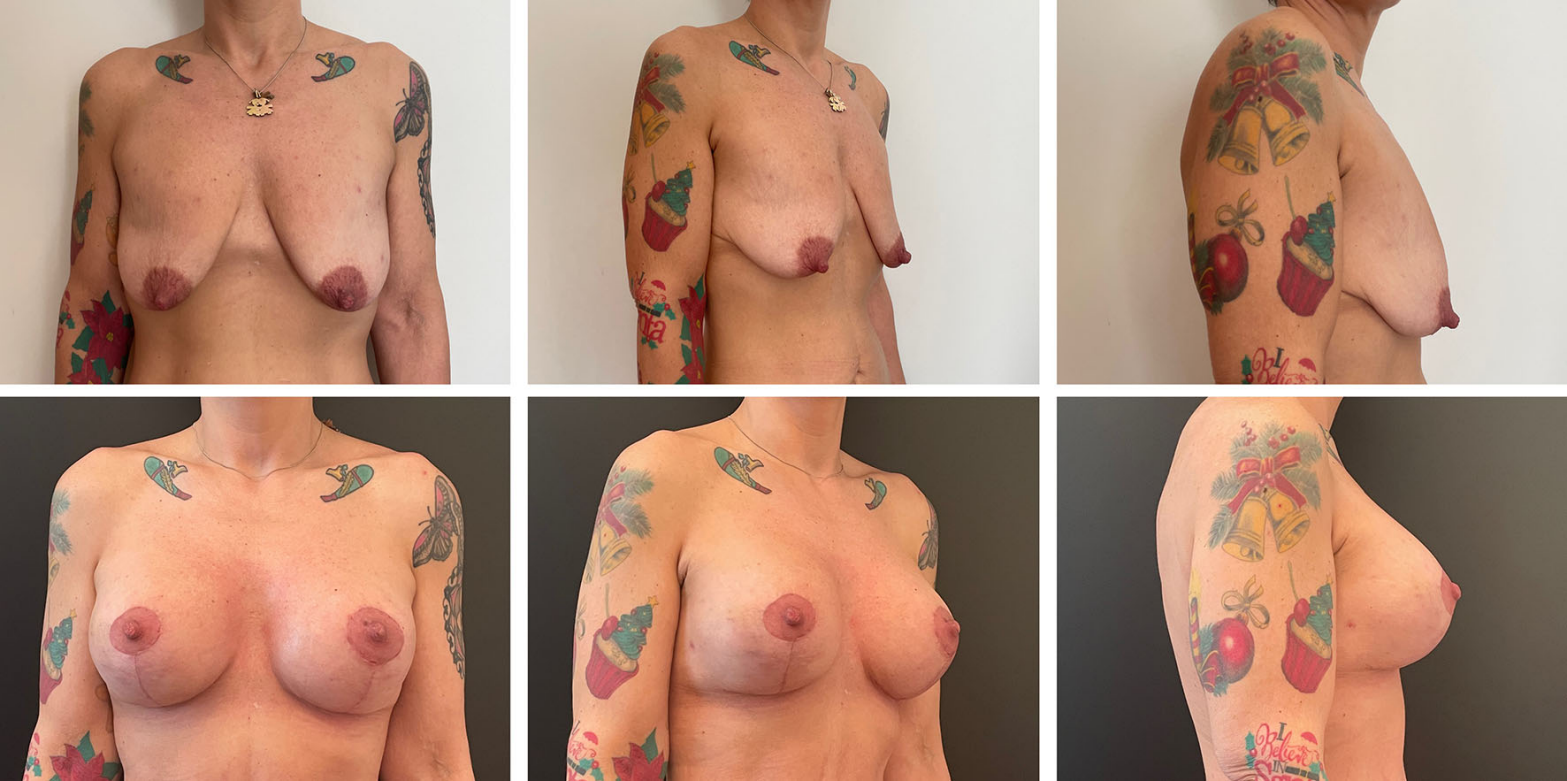
Preoperative (above) and postoperative results at one year (below) of a sample case for Group B

**Fig. 5:**
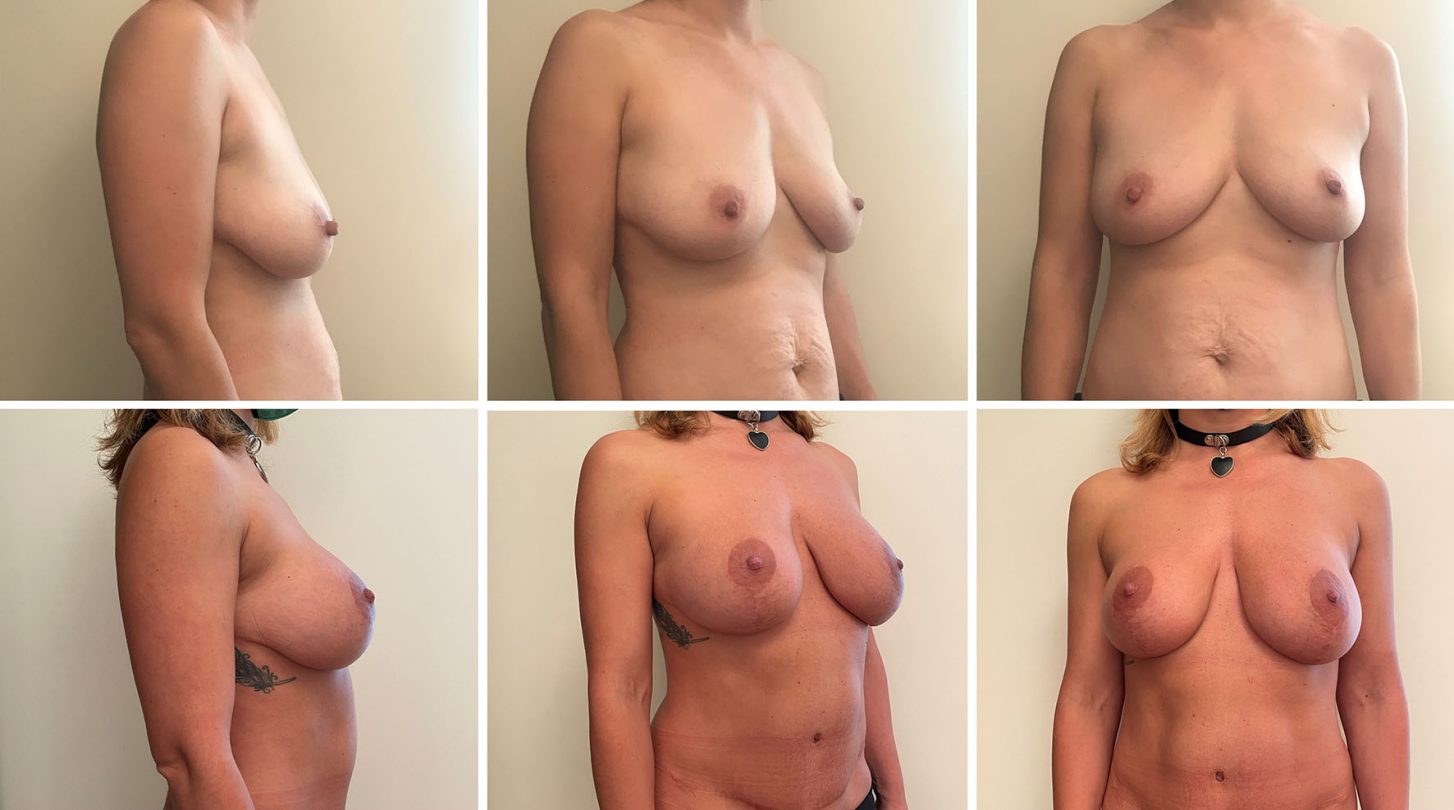
Preoperative (above) and postoperative results at 10 years (below) of a sample case for Group B

Complications were registered in ten patients (9.3%) in Group A (two wound breakdowns at T with implant exposure and eight wound dehiscences without implant exposure), six of which required surgical revision. In contrast, only three patients (2.8%) in Group B reported a complication, which was wound dehiscence without implant exposure in all cases. None of the dehiscence required surgical revision. One dehiscence in Group A and one in Group B occurred in active smokers. The difference between complication and revision rates in the two groups was statistically significant (*p* = .045 and .035, respectively). Patient characteristics and outcomes are summarized in Table 1. The results of pre- and postoperative day 30 of the case shown in the surgical technique paragraph are shown in Figure 2. Example cases at 1 year follow-up are shown in Figure 3 for Group A and in Figure 4 for Group B. Example case of a 10 year follow-up for Group B is shown in Figure 5. A complete list of patients and their data are reported in Table 2.

**Table 1:** Patients characteristics

	Group A	Group B	*P* value
	Wise pattern	Chimeric mastopexy	
*N*	107	109	
Median age (years)	28	25	NS
Smokers (*N*)	12	8	NS
Ptosis			
Grade II (*n*)	25	33	NS
Grade III (*n*)	82	76	NS
Implant			
Round (*N*)	21	22	NS
Shaped (*n*)	86	87	NS
Mean volume (CC)	290	300	NS
SN-N distance (cm)			
Mean pre	25	25	NS
Mean post	19	20	NS
Complications (*n*)	10	3	< .05
Dehiscence (*n*)	8	3	NS
Exposure (*n*)	2	0	NS
Revision (*n*)	6	0	< .05
FU (months)	46	43	NS

**Table 2:** Complete patients data

							S-N distance		N-IMF distance						
*N*	Age	Smoke	Ptosis	Mastopexy	Impl. type	Impl. volume	Pre	Post	Delta	Pre2	Post3	Delta	Complications	Reoperation	FU
6	18		II	Wise	Shaped	320	24	21	3	13	7	6			25
10	24		II	Wise	Round	275	23	20	3	13	8	5			62
11	29		II	Wise	Shaped	335	24	21	3	13	7	6			46
16	32		II	Wise	Shaped	245	23	20	3	13	8	5			53
28	27		II	Wise	Shaped	285	25	21	4	13	9	4			25
30	27		II	Wise	Shaped	235	23	21	2	11	9	2	Exposure	Exchange	58
40	33		II	Wise	Shaped	245	23	20	3	12	7	5			33
41	30		II	Wise	Shaped	315	23	19	4	12	8	4			63
43	31	10	II	Wise	Round	275	24	21	3	13	8	5			63
44	41		II	Wise	Shaped	265	24	20	4	10	9	1			66
53	41		II	Wise	Shaped	355	23	19	4	11	8	3			56
57	19		II	Wise	Shaped	270	25	21	4	13	8	5	Dehiscence	Revision	45
58	21	14	II	Wise	Shaped	305	24	20	4	11	7	4			56
60	25		II	Wise	Shaped	285	23	21	2	10	9	1			63
1	26		III	Wise	Shaped	320	26	20	6	10	8	2			69
2	39		III	Wise	Shaped	330	23	18	5	10	8	2			67
3	27		III	Wise	Shaped	345	25	20	5	10	9	1			46
4	26		III	Wise	Shaped	285	24	19	5	11	7	4			53
5	22		III	Wise	Shaped	260	24	18	6	10	9	1			46
7	21		III	Wise	Shaped	280	25	19	6	12	9	3			44
8	20	12	III	Wise	Shaped	310	26	18	8	13	7	6	Dehiscence	Revision	51
9	22		III	Wise	Shaped	355	27	18	9	10	8	2			26
12	22		III	Wise	Shaped	260	24	19	5	13	8	5			56
13	21		III	Wise	Round	275	25	19	6	13	7	6			55
14	25		III	Wise	Shaped	330	28	20	8	12	8	4			49
15	28		III	Wise	Round	250	26	21	5	11	8	3			44
17	26		III	Wise	Shaped	230	26	19	7	13	9	4			60
18	24	5	III	Wise	Shaped	315	25	20	5	10	7	3			22
19	31		III	Wise	Shaped	295	23	18	5	12	8	4			31
20	19		III	Wise	Shaped	255	26	20	6	10	8	2			20
21	31		III	Wise	Round	250	25	20	5	13	8	5	Dehiscence	Revision	60
22	23		III	Wise	Shaped	285	26	20	6	10	8	2			59
23	21		III	Wise	Shaped	230	23	18	5	11	9	2			53
24	23		III	Wise	Shaped	290	27	21	6	12	8	4			69
25	31		III	Wise	Round	325	25	19	6	13	9	4			67
26	22		III	Wise	Round	250	25	18	7	10	9	1			40
27	27	8	III	Wise	Shaped	315	28	21	7	13	7	6			24
29	40		III	Wise	Shaped	240	26	21	5	12	8	4			70
31	26		III	Wise	Shaped	315	26	20	6	10	9	1	Dehiscence		28
32	29		III	Wise	Shaped	345	25	19	6	13	9	4			35
33	26		III	Wise	Shaped	235	24	19	5	13	9	4			52
34	32		III	Wise	Shaped	290	26	18	8	12	9	3			46
35	37		III	Wise	Shaped	290	28	19	9	12	7	5			18
36	26		III	Wise	Shaped	340	26	21	5	13	7	6			67
37	27		III	Wise	Shaped	320	25	18	7	12	8	4			46
38	27		III	Wise	Round	300	23	18	5	12	9	3			50
39	19		III	Wise	Shaped	305	26	19	7	11	9	2			61
42	36	10	III	Wise	Shaped	240	25	20	5	12	8	4			21
45	28		III	Wise	Shaped	280	26	20	6	13	7	6			52
46	35		III	Wise	Round	375	25	20	5	11	8	3			29
47	41		III	Wise	Shaped	270	26	20	6	10	8	2			49
48	40		III	Wise	Shaped	235	24	19	5	11	8	3			67
49	32		III	Wise	Shaped	270	26	21	5	11	9	2			58
50	27		III	Wise	Round	250	26	18	8	13	7	6			57
51	18		III	Wise	Shaped	335	26	21	5	11	7	4	Dehiscence	None	40
52	35	14	III	Wise	Round	300	26	18	8	10	8	2			41
54	22		III	Wise	Shaped	315	27	19	8	12	9	3			25
55	20		III	Wise	Shaped	295	25	20	5	12	8	4			19
56	23		III	Wise	Shaped	310	26	18	8	11	7	4			22
59	23		III	Wise	Shaped	335	28	20	8	13	8	5			40
61	32		III	Wise	Shaped	290	26	18	8	11	7	4			49
62	35		III	Wise	Shaped	280	26	18	8	12	7	5			21
172	22	6	III	Wise	Shaped	230	27	19	8	11	7	4			39
173	19		III	Wise	Shaped	240	23	18	5	13	8	5			47
174	19		III	Wise	Round	325	27	21	6	11	7	4			51
175	24		III	Wise	Shaped	355	27	21	6	11	7	4			20
176	30		III	Wise	Shaped	335	25	18	7	12	9	3			19
177	25		III	Wise	Shaped	335	23	18	5	11	7	4			63
178	37		II	Wise	Shaped	365	23	20	3	10	8	2			61
179	18	8	II	Wise	Shaped	265	23	21	2	13	7	6			62
180	32		III	Wise	Shaped	330	26	18	8	11	7	4			69
181	37		II	Wise	Shaped	255	22	19	3	12	7	5			32
182	31		III	Wise	Shaped	255	26	19	7	11	8	3			37
183	37		III	Wise	Round	275	26	19	7	13	7	6	Dehiscence		35
184	34		III	Wise	Shaped	360	26	21	5	13	9	4			51
185	36		II	Wise	Shaped	330	23	19	4	12	9	3			52
186	25	6	III	Wise	Round	250	23	18	5	13	9	4			43
187	24		III	Wise	Round	300	26	19	7	13	7	6			53
188	30		III	Wise	Shaped	240	25	19	6	13	9	4			54
189	29		III	Wise	Round	250	24	18	6	11	8	3			24
190	35		III	Wise	Shaped	285	24	19	5	10	7	3			56
191	29		III	Wise	Shaped	320	27	21	6	13	7	6			54
192	26		III	Wise	Round	250	29	20	9	12	9	3			49
193	22		III	Wise	Shaped	240	26	18	8	12	7	5			50
194	33		III	Wise	Shaped	340	27	20	7	12	9	3	Dehiscence	Revision	36
195	34		III	Wise	Shaped	365	23	18	5	11	9	2			59
196	34		II	Wise	Shaped	370	23	19	4	13	8	5			66
197	27		II	Wise	Shaped	310	24	20	4	11	7	4			40
198	20		II	Wise	Round	275	23	21	2	10	9	1			33
199	30	8	II	Wise	Shaped	345	23	19	4	12	8	4			70
200	21	10	II	Wise	Shaped	280	22	19	3	11	8	3			34
201	29		III	Wise	Shaped	315	27	19	8	12	8	4			19
202	18		III	Wise	Shaped	235	27	21	6	13	7	6			70
203	29		III	Wise	Round	325	23	18	5	11	8	3			69
204	30		III	Wise	Shaped	355	26	21	5	12	7	5			53
205	34		III	Wise	Shaped	270	24	19	5	10	9	1			19
206	32		III	Wise	Shaped	305	26	20	6	12	7	5	Exposure	Exchange	25
207	29		II	Wise	Shaped	280	23	19	4	13	7	6			67
208	34		III	Wise	Round	275	28	21	7	12	9	3			42
209	25		III	Wise	Round	375	27	20	7	10	7	3			64
210	29		II	Wise	Shaped	245	25	21	4	10	7	3			64
211	32		III	Wise	Shaped	240	24	19	5	10	9	1			58
212	23		III	Wise	Shaped	295	24	18	6	10	9	1	Dehiscence		55
213	34		III	Wise	Shaped	345	28	21	7	13	9	4			49
214	33		III	Wise	Shaped	255	24	18	6	10	7	3			49
215	30		III	Wise	Shaped	260	25	20	5	10	9	1			27
216	34		III	Wise	Shaped	315	28	20	8	11	9	2			26
63	20		II	Hybrid	Shaped	305	23	19	4	12	7	5			52
66	26		II	Hybrid	Shaped	255	23	19	4	11	7	4			34
71	22		II	Hybrid	Shaped	355	25	21	4	12	9	3			36
72	21		II	Hybrid	Shaped	370	25	21	4	11	9	2			69
75	30		II	Hybrid	Shaped	310	24	20	4	10	8	2			39
81	22	10	II	Hybrid	Shaped	285	23	20	3	13	8	5	Dehiscence	None	36
88	29		II	Hybrid	Round	350	23	20	3	13	7	6			63
89	26		II	Hybrid	Round	300	23	19	4	12	7	5			51
90	22		II	Hybrid	Shaped	280	23	19	4	12	7	5			23
93	27		II	Hybrid	Shaped	255	24	20	4	13	8	5			61
94	22	12	II	Hybrid	Shaped	295	23	19	4	13	9	4			66
101	24		II	Hybrid	Shaped	360	23	19	4	11	9	2			32
102	28		II	Hybrid	Shaped	305	24	21	3	10	7	3			45
107	25		II	Hybrid	Shaped	355	24	21	3	12	7	5			63
111	20		II	Hybrid	Shaped	230	23	21	2	13	8	5			26
117	20		II	Hybrid	Round	350	25	21	4	12	7	5			58
120	29	8	II	Hybrid	Shaped	305	25	21	4	13	7	6			49
121	22	8	II	Hybrid	Shaped	295	23	19	4	10	8	2			66
122	21		II	Hybrid	Round	325	23	19	4	13	8	5			30
124	24		II	Hybrid	Round	250	23	21	2	12	7	5			54
129	30		II	Hybrid	Shaped	345	23	19	4	10	7	3			44
130	28		II	Hybrid	Shaped	260	23	20	3	10	9	1			62
131	25		II	Hybrid	Round	375	24	21	3	10	9	1			23
134	30		II	Hybrid	Shaped	370	24	20	4	13	8	5			35
135	29		II	Hybrid	Shaped	360	23	21	2	10	7	3			67
145	25		II	Hybrid	Shaped	285	23	20	3	13	8	5			55
64	22		III	Hybrid	Round	350	27	20	7	11	7	4			36
65	20		III	Hybrid	Shaped	340	25	19	6	12	9	3			29
67	24		III	Hybrid	Shaped	290	28	19	9	11	8	3			41
68	20		III	Hybrid	Shaped	270	24	18	6	10	8	2			32
69	23		III	Hybrid	Shaped	315	28	20	8	12	9	3			18
70	28		III	Hybrid	Shaped	340	26	21	5	11	8	3			22
73	25		III	Hybrid	Shaped	230	27	18	9	12	7	5	Dehiscence	None	59
74	23		III	Hybrid	Shaped	230	23	18	5	11	8	3			22
76	28		III	Hybrid	Shaped	320	26	18	8	10	7	3			34
77	29		III	Hybrid	Round	250	27	19	8	13	8	5			18
78	26		III	Hybrid	Shaped	355	27	20	7	10	8	2			64
79	23		III	Hybrid	Shaped	305	27	21	6	12	7	5			47
80	21	14	III	Hybrid	Round	250	25	19	6	10	9	1			37
82	25		III	Hybrid	Round	325	25	20	5	13	9	4			42
83	23		III	Hybrid	Shaped	330	26	19	7	12	7	5			54
84	31		III	Hybrid	Shaped	360	27	21	6	12	8	4			24
85	30		III	Hybrid	Shaped	330	24	18	6	11	9	2			54
86	28		III	Hybrid	Shaped	335	26	21	5	12	8	4			32
87	25		III	Hybrid	Shaped	230	27	18	9	11	8	3			53
91	20		III	Hybrid	Round	300	26	21	5	10	9	1			26
92	24		III	Hybrid	Shaped	305	23	18	5	13	8	5			50
95	30		III	Hybrid	Shaped	370	27	21	6	12	8	4			27
96	23		III	Hybrid	Round	350	28	19	9	12	8	4			36
97	30		III	Hybrid	Shaped	260	26	20	6	10	8	2			37
98	25		III	Hybrid	Shaped	355	24	19	5	11	7	4			51
99	30		III	Hybrid	Shaped	295	28	20	8	13	8	5			63
100	23		III	Hybrid	Shaped	260	26	18	8	13	7	6			65
103	26		III	Hybrid	Shaped	290	26	20	6	13	7	6			51
104	21		III	Hybrid	Shaped	255	25	18	7	12	8	4			70
105	31		III	Hybrid	Round	325	27	20	7	10	8	2			63
106	23		III	Hybrid	Shaped	230	25	19	6	10	8	2			68
108	27		III	Hybrid	Shaped	295	24	19	5	12	8	4			19
109	28		III	Hybrid	Shaped	245	25	18	7	12	9	3			68
110	29		III	Hybrid	Shaped	230	27	20	7	12	7	5			66
112	31	12	III	Hybrid	Shaped	230	27	21	6	11	8	3			41
113	20		III	Hybrid	Shaped	295	25	20	5	10	9	1			20
114	29		III	Hybrid	Round	375	28	21	7	12	8	4			42
115	20		III	Hybrid	Shaped	340	28	19	9	11	7	4			26
116	24		III	Hybrid	Shaped	340	24	18	6	12	9	3	Dehiscence	None	65
118	25		III	Hybrid	Shaped	265	27	18	9	13	7	6			47
119	30		III	Hybrid	Shaped	265	25	19	6	11	9	2			64
123	28		III	Hybrid	Round	275	24	18	6	11	9	2			67
125	29		III	Hybrid	Shaped	280	25	18	7	11	9	2			62
126	22		III	Hybrid	Shaped	265	24	18	6	12	7	5			61
127	32		III	Hybrid	Shaped	335	27	19	8	11	7	4			53
128	20		III	Hybrid	Shaped	230	26	21	5	12	7	5			42
132	31		III	Hybrid	Shaped	290	28	20	8	12	8	4			27
133	22		III	Hybrid	Round	250	27	21	6	10	7	3			53
136	28		III	Hybrid	Shaped	230	24	18	6	10	9	1			61
137	21		III	Hybrid	Round	325	23	18	5	11	8	3			59
138	28		III	Hybrid	Shaped	355	28	21	7	13	9	4			19
139	26		III	Hybrid	Shaped	285	27	19	8	11	8	3			49
140	31		III	Hybrid	Shaped	265	26	21	5	10	7	3			36
141	30		III	Hybrid	Shaped	320	26	18	8	10	8	2			47
142	21		III	Hybrid	Shaped	295	28	20	8	12	7	5			18
143	20		III	Hybrid	Shaped	235	24	18	6	13	8	5			42
144	25		III	Hybrid	Shaped	320	26	21	5	13	9	4			23
146	27		III	Hybrid	Shaped	255	28	21	7	13	7	6			32
147	26		II	Hybrid	Shaped	360	23	19	4	13	8	5			27
148	20		III	Hybrid	Shaped	365	23	18	5	10	7	3			48
149	31	5	III	Hybrid	Shaped	285	23	18	5	13	9	4			59
150	21		II	Hybrid	Shaped	370	24	21	3	12	7	5			34
151	25		III	Hybrid	Shaped	330	24	19	5	12	9	3			68
152	23		III	Hybrid	Shaped	340	26	20	6	12	7	5			31
153	23		II	Hybrid	Shaped	285	25	21	4	10	7	3			28
154	31		III	Hybrid	Shaped	345	25	20	5	13	9	4			39
155	21		III	Hybrid	Shaped	335	29	20	9	13	7	6			64
156	29		III	Hybrid	Round	375	25	18	7	11	9	2			40
157	28		III	Hybrid	Shaped	280	28	21	7	10	8	2			69
158	22		III	Hybrid	Round	375	28	20	8	13	8	5			44
159	30		III	Hybrid	Shaped	260	29	21	8	10	8	2			59
160	32		III	Hybrid	Shaped	240	24	18	6	12	8	4			22
161	23		III	Hybrid	Round	300	29	20	9	11	7	4			28
162	26		III	Hybrid	Shaped	240	28	20	8	10	7	3			42
163	26		II	Hybrid	Shaped	365	23	20	3	12	8	4			29
164	29		III	Hybrid	Shaped	280	29	21	8	13	7	6			22
165	30		III	Hybrid	Round	300	28	20	8	13	8	5			47
166	20		II	Hybrid	Shaped	310	24	20	4	10	9	1			43
167	20		II	Hybrid	Shaped	265	23	20	3	10	7	3			30
168	21	10	III	Hybrid	Shaped	295	24	18	6	12	8	4			38
169	29		III	Hybrid	Shaped	345	26	19	7	11	9	2			46
170	28		III	Hybrid	Shaped	290	25	18	7	11	9	2			29
171	24		II	Hybrid	Round	300	24	21	3	11	8	3			26

## Discussion

Mastopexy and augmentation mammoplasty have opposite surgical goals, with the increase in the breast volume contrasting the skin reduction. Consequently, single-stage procedures reported relatively high frequencies of complications, among which wound dehiscence with implant exposure represents the most serious local complication.

Khanavin et al. [18] performed a systematic review and meta-analysis of the outcomes of single-stage augmentation mastopexy from 23 retrospective cohort studies. They reported a pooled complication rate of 13.1% (6.7–21.3% CI) and a pooled reoperation rate of 10.65% (6.7–15.4% CI). The inverted-T resulted in a lower rate of ptosis recurrence (3.2%). A major limitation of their study was the evident heterogeneity among studies, both in quality and level of evidence; nevertheless, their study is the only meta-analysis available to date on single-stage augmentation mastopexy. With our modifications, combining the approach of three different breast operations, we lowered the complication rate from 9.3 to 3% and the revision rate from 5.6% to none. By integrating techniques from three distinct breast operations, we reduced the complication rate from 9.3 to 3% and eliminated the revision rate, which was 5.6% in Group A. Interestingly, even if three wound dehiscences were registered in the modified technique group, none of them resulted in implant exposure or needed reoperation.

Stevens et al. [9] reported on 615 consecutive patients undergoing single-stage mastopexy. Implant infection with explanation occurred in 0.8%, poor scarring in 5.7%, and wound-healing problems in 2.9% of patients. The global revision rate was 16.9%. The major limitation of their study is the extreme variability of their cohort: they included different procedures (both primary and secondary mastopexy), implant materials (silicone and saline), surgical techniques (circumareolar, vertical, and inverted-T), and pocket location (both sub glandular and submuscular). Messa et al. [10] reported similar outcomes in their 1183 consecutive single-stage augmentation mastopexy cohort. Although, they also included circumareolar, vertical, and inverted-T techniques. In contrast, in our study, we included only a specified type of augmentation mastopexy, reporting a 2.8% of complications with no revisions.

Sanniec et al. [3] reported one of the largest single-operator case series in the literature, with 251 single-stage augmentation mastopexies. In their study, the mastopexy technique was like our inverted-T, including the preservation of the inferior dermal flap at the T; though, the elevation of the two parenchymal pillars was conducted at full thickness, connecting the skin incision directly to the implant pocket. They reported 14% of total complications, 3.6% revisions, and 0.8% implant removal. On the contrary, our modified technique achieved sensibly lower rates and no revisions.

Our modified chimeric technique reunites the advantages of three different approaches in breast surgery. The inframammary incision and submuscular placement of the implant reduce the risk of infection and capsular contracture [19]; the inferior pole de-epithelized flap, beveling the access of the implant pocket from the inframammary part of the inverted-T wound, offers vascularized coverage to the inferior profile of the implant and avoids direct exposure in the case of a wound breakdown. This was partially derived from the Balcony technique described by De Vita et al. [20]. Finally, subcutaneous dissection with elevation of thin and pliable medial and lateral pillar flaps, derived from the peri-areolar and vertical mastopexy techniques, optimizes skin re-draping, and maximizes control over nipple position and lower pole height.

The main limitation of this study stands in the retrospective nature, which does not allow a strict control of confounding biases, even if they had been reduced by the cohort matching, the single operator procedures, the equality among the types of implants, and the degree of breast lift. A prospective study might help confirming that the described technique is able to reduce the rate of complication and revision in augmentation mastopexy.

## Conclusions

Our chimeric technique of single-stage augmentation mastopexy improves the safety profile of this intervention, reducing overall complications and minimizing the risk for wound dehiscence and implant exposure.

## Supplementary Information

Below is the link to the electronic supplementary material.

This video shows the preoperative planning and design for our technique of single-stage augmentation mastopexy. (MP4 61102 KB)

This video shows an intraoperative technical tip. After implant placement and de-epithelization, the medial and lateral pillars are elevated. The central sling of dermis has to be incised, in order to facilitate the lifting of the NAC. (MP4 42476 KB)
